# 

**Published:** 1881-12

**Authors:** 


VOL. II. DECEMBER, 1881. NO. 12.

—: THE :—

### Independent Practitioner:

A MONTHLY JOURNAL,

S. G. O. INDEXED

DEVOTED TO

***MEDICAL, SURGICAL, OBSTETRICAL, DENTAL, HYGIENICAL AND POPULAR SCIENCES.***

EDITED BY

BASIL M. WILKERSON, D. D. S., M. D.

**"Altera poscit opem res, et conjurat amice."**

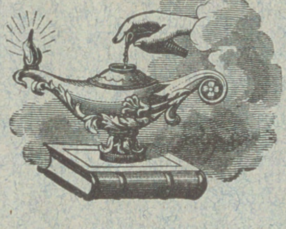

NEW YORK:

*Published by B. M. WILKERSON, Proprietor,*

Office, 20 Courtlandt Street.

**$3.00 Per Annum in Advance, - - - - Single Copies 30 Cents.**

Entered at the Post Office, New York City, as Second-Class Matter.

